# The Independent and Cumulative Effects of Sibling and Peer Bullying in Childhood on Depression, Anxiety, Suicidal Ideation, and Self-Harm in Adulthood

**DOI:** 10.3389/fpsyt.2019.00651

**Published:** 2019-09-24

**Authors:** Slava Dantchev, Matthew Hickman, Jon Heron, Stanley Zammit, Dieter Wolke

**Affiliations:** ^1^Department of Psychology, University of Warwick, Coventry, United Kingdom; ^2^Faculty of Psychology, University of Vienna, Vienna, Austria; ^3^Population Health Sciences, Bristol Medical School, University of Bristol, Bristol, United Kingdom; ^4^Medical Research Council Centre for Neuropsychiatric Genetics and Genomics, Cardiff University, Cardiff, United Kingdom; ^5^Division of Mental Health & Wellbeing, Warwick Medical School, University of Warwick, Coventry, United Kingdom

**Keywords:** siblings, bullying, depression, anxiety, self-harm, ALSPAC

## Abstract

Sibling and peer bullying are reported as the most frequent forms of violence experienced across childhood. There is now ample evidence indicating an association between sibling and peer bullying, with those reporting sibling bullying at an increased risk of peer bullying. While there is convincing evidence of a causative association between peer bullying and a range of mental health outcomes, sibling bullying continues to receive far less attention. The aim of this study was to explore whether sibling bullying roles (non-involved, victim, bully-victim, bully) in middle childhood were independently associated with clinical diagnoses of depression and anxiety and reports of suicidal ideation and self-harm in early adulthood. We further tested whether there was a cumulative relationship between involvement in sibling and peer bullying victimization. This study was based on up to 3,881 youth from the Avon Longitudinal Study of Parents and Children, a prospective birth-cohort based in the United Kingdom. Sibling and peer bullying was assessed *via* self-report when youth were 12 years of age, while depression, anxiety, suicidal ideation, and self-harm were assessed *via* self-administered computerized interviews at 24 years of age. Involvement as a sibling bully-victim was associated with clinical diagnosis of depression (OR = 1.91, 95% CI: 1.33–2.72), while sibling victims were at increased odds of both suicidal ideation (OR = 1.52; 95% CI, 1.16–1.98) as well as suicidal self-harm (OR = 2.20, 95% CI, 1.36–3.58) in early adulthood, even after accounting for concurrent peer bullying and a range of other pre-existing childhood confounders. Sibling and peer bullying were further associated in a homotypic manner. A dose–response relationship of bullying in the home and school across mental health outcomes was found. Youth victimized by both their siblings and peers displayed the highest odds of developing clinical depression, suicidal ideation, and self-harm. Children bullied at home and at school had no safe place to escape the bullying and torment. Our findings highlight the need for intervention studies tailored toward reducing sibling bullying, as these may hold large promise for alleviating a range of adverse outcomes, including the prevention of peer bullying, which may be contingent on early bullying experiences in the home environment.

## Introduction

Bullying occurs in social situations where a person cannot choose the peers they are interacting with and are instead “caged” together in an environment such as a school classroom or the work place. The prime example in which a child becomes “caged” together with others and is unable to leave or escape this environment is with siblings, in the family. Thus, repeated unwanted aggressive behavior by a sibling that intends to inflict harm either physically, psychologically, or socially and involves an imbalance of power is called sibling bullying (1). Peer and sibling violence have been reported as the most frequent forms of violence experienced across childhood exceeding any violence by adults (2, 3). While there is increased recognition of the adverse effects of peer bullying, sibling bullying is still largely perceived as normative behavior across development (4, 5) and continues to receive far less attention as opposed to peer bullying (6).

In the peer literature, there is now convincing evidence of a causative association between peer bullying and depression, anxiety and self-harm (7–11). The general consensus from the literature suggests that peer victims are at increased risk of internalizing disorders, whereas peer bullies are at increased risk of externalizing disorders, with peer bully-victims suffering the greatest adult consequences, including both more internalizing and externalizing disorders (12). Findings from meta-analyses indicate that involvement in any peer bullying increases the risk of suicidal ideation and behavior (13), whereas peer victimization, in contrast to peer bullying perpetration, has specifically been associated with an elevated risk of anxiety disorders, depression, self-harm, suicidal ideation, and attempts (10, 11), even after accounting for other major childhood risk factors, trauma and genetic liability (14). In contrast, research on the adverse outcomes of sibling bullying is still in its infancy. There is an emerging body of research linking sibling bullying in childhood to a range of internalizing and mental health problems both concurrently and prospectively (6, 15–22).

To the best of our knowledge there has only been one previous prospective study exploring the relationship between sibling bullying and the risk of depression, anxiety, and self-harm. Using a sample of over 6,900 children from the Avon Longitudinal Sample of Parents and Children (ALSPAC) in the UK, Bowes et al. (16) found that sibling bullying victimization increased the risk of depression, anxiety, and self-harm two-fold, with results remaining similar in strength for depression and self-harm even after accounting for a range of childhood confounders. Replications of such findings have been limited to cross-sectional designs. For instance, Coyle et al. (6) found that sibling bullying victimization was associated with increased risk for anxiety and depressive symptoms, over and beyond the experience of peer bullying in a sample of 372 US students. Similarly, Bar-Zomer and Klomek (15) reported an association between involvement in any kind of sibling bullying (victimization and perpetration) and greater risk for depression and suicidal ideation, using a sample of 279 Israeli students. In order to consolidate these findings, future replications are needed using prospective studies in large samples.

A further caveat of the literature is the lack of studies exploring the differential outcomes according to sibling bullying status groups. Children are typically classified into one of four bullying groups: non-involved, victims, bully-victims or bullies. These classifications are important, as children have been found to experience differential mental health and behavioral outcomes depending on their role in sibling bullying (17, 18, 23). In the peer literature, bully-victims have been reported to be at a particular risk of developing mental health problems, especially in relation to depression, anxiety and suicidality (7, 9, 24, 25). Research exploring differential outcomes in relation to sibling bullying roles is scant. Only a handful of studies suggest a similar trend for sibling bully-victims for internalizing problems (18), psychotic disorders (23) as well as high-risk behavior (17). There are however, no previous studies that have tested whether sibling bullying involvement is differentially associated with depression, anxiety, suicidal ideation or self-harm depending on the sibling bullying role. Previous research has either focused solely on sibling bullying victimization (6, 16) or has combined victimization and perpetration into the same underlying construct, without teasing out the mutually independent groups (15). Hence, there is a pressing need for prospective studies to explore the relationship between involvement in different sibling bullying roles and depression, anxiety, suicidal ideation, and self-harm.

Finally, there is now robust evidence indicating an association between bullying across the home and school context, with those children reporting sibling bullying found at an increased risk of peer bullying involvement (26–29). The association has been, where investigated, found to be homotypic with victims of sibling bullying also more likely to be victims in peer bullying and bullies and bully/victims more often perpetrators or bully/victims in peer relationships (27). Furthermore, experimental research indicates that those involved in sibling aggression in toddlerhood are more likely to also use aggressive strategies with peers 18 months later (30). Thus, sibling bullying appears to precede peer bullying involvement. In other words, those children who are victims at home are more likely to be victims at school and may therefore have no safe space to escape the bullying. Hence, the negative effects of trauma have been reported to be additive (14). Indeed, there is some evidence suggesting a cumulative risk of involvement in both sibling and peer bullying with a range of adverse outcomes including clinically significant behavioral outcomes and mental health distress (19, 20, 26, 29). Findings using longitudinal data have furthermore found that children involved in bullying across the sibling and peer context are at increased risk of psychotic disorder (23) as well as high-risk behavior (17) lasting until early adulthood. It is unknown, whether the there is a similar longitudinal dose-effect relationship between involvement in both sibling and peer bullying in childhood with depression, anxiety, suicidal ideation or self-harm into early adulthood.

In summary, there is an urgent need for prospective longitudinal studies that investigate both sibling and peer bullying and that distinguish the differential effects of involvement in sibling bullying as victims, bully-victims, or bullies compared to non-involved children. Such prospective designs may further help determine the individual and joint additive or moderating effects of sibling versus peer bullying on depression, anxiety, suicidal ideation, and self-harm, while controlling for pre-existing mental health problems and other confounders at the same time.

This study builds on previous work conducted by Bowes et al. (16) incorporating a new wave of data collection on clinical diagnosis of depression and anxiety disorder, suicidal ideation, and self-harm a further 6 years on at 24 years of age. We addressed the following research question: (1) Are sibling bullying roles (non-involved, victim, bully-victim, bully) differentially associated with depression, anxiety, suicidal ideation, and self-harm in late adolescence (at 18 years) and early adulthood (at 24 years)? (2) Does sibling bullying predict depression, anxiety, suicidal ideation, and self-harm in early adulthood over and above peer bullying? (3) Is there a cumulative relationship between sibling and peer bullying in middle childhood and depression, anxiety, suicidal ideation, and self-harm in early adulthood?

## Method

### Study Design

This study draws on data from the Avon Longitudinal Study of Parents and Children (ALSPAC). Pregnant women residing in Avon, UK with expected dates of delivery from 1^st^ April 1991 to 31^st^ December 1992 were invited to take part in the study. The initial number of pregnancies enrolled in the study was 14,541, which included all women who had completed and returned at least one questionnaire or attended one of the ‘Children in Focus’ clinic sessions. There were a total of 14,062 live births and 13,988 children were alive at 1 year of age. Participant data has been collected on the mothers, fathers and children from early pregnancy and has been measured *via* questionnaires and regular clinic visits. Children have been invited to attend a total of 10 assessment clinics, including face-to-face interviews and psychological and physical tests, from age 7 years onward. Detailed reports on the recruitment and enrollment processes of the mother and child cohort are available in the cohort profiles (31–33). Part of this data was collected using REDCap (https://projectredcap.org/resources/citations/). Please note that the study website contains details of all the data that is available through a fully searchable data dictionary and variable search tool (http://www.bristol.ac.uk/alspac/researchers/our-data/).

Ethical approval for the study was obtained from the ALSPAC Ethics and Law Committee (IRB No. 00003312) and the local research ethics committees (Bristol and Weston Health Authority, Southmead Health Authority and Frenchay Health Authority). Informed consent for the use of data collected *via* questionnaires and clinics was obtained from participants following the recommendations of the ALSPAC Ethics and Law Committee at the time.

### Sample

**Figure 1:**
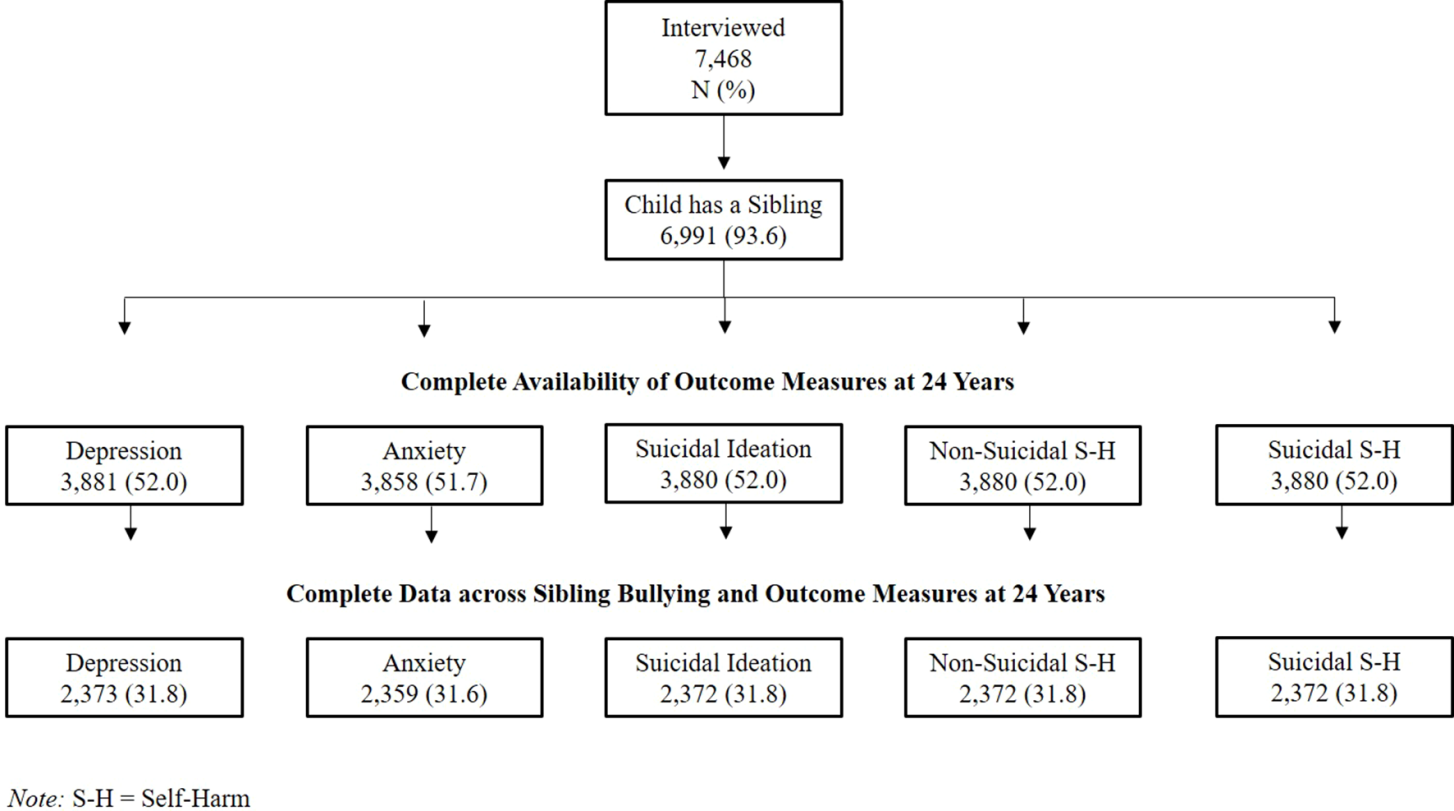
Flowchart of sample size distribution across outcome measures at 24 years.

At the 12-year assessment questionnaires were sent out to 11,132 eligible families of which 7,505 (67.4%) were completed and returned. Children without siblings (N = 477; 6.4%) were excluded, resulting in a starting sample of 6,928 youth who completed detailed questions on sibling bullying when they were on average 12.1 years old. Outcome data was available for up to 3,881 adolescents at 24 years. We further omitted participants who presented any psychiatric diagnosis (DSM-IV Axis I diagnoses; N = 475) prior to our measure of exposure (sibling bullying) throughout our all analysis. An illustration of our complete data sample across each outcome measure is provided in **Figure 1**.

### Assessment of Sibling Bullying

Sibling bullying was assessed *via* an adapted version of the Olweus Bullying Questionnaire (18, 34) when children were approximately 12 years of age. Youth were first told that they would be asked a series of questions about sibling bullying explaining that this meant when a brother or sister ‘tries to upset [them] by saying nasty and hurtful things, or completely ignores [them] from their group of friends, hits, kicks, pushed, or shoves [them] around, tells lies or makes up false rumors about [them]’. Youth were asked to report whether they had ever been bullied by a sibling at home in the past 6 months *via* a 5-point Likert-scale (0 = never; 1 = only ever once or twice; 2 = 2 or 3 times a month; 3 = about once a week; 4 = several times a week). Youth were then asked to report on their experience of specific types of sibling bullying *via* 6 items, including physical (hitting, kicking or shoving), psychological (being called nasty names; making fun of), property-based (having belongings damaged or taken) and social (excluding; telling lies or spreading rumors), using the same 5-point Likert scale. In order to determine the frequency of sibling bullying victimization, the highest reported frequency across all items was used. Youth were also asked about sibling bullying perpetration, using the same method described above. The internal consistency (Cronbach’s alpha) across victimization (a = .80) and perpetration items (a = .74) was found to be high.

Youth were also classified into one of four sibling bullying roles (non-involved, victim, bully-victim, bully) using a cut-off of frequent sibling bullying (at least once a week). Youth reporting no bullying at all *or* bullying experiences less than once a week were categorized as ‘non-involved’. Youth reporting frequent victimization only were categorized as ‘victims’. Youth reporting frequent perpetration only were categorized as ‘bullies. Youth reporting *both* frequent victimization and perpetration were categorized as ‘bully-victims’.

### Assessment of Peer Bullying

Peer bullying was assessed *via* a modified version of the Bullying and Friendship Interview Schedule (35) when children were 12 years of age. Youth were told that they would be asked a few questions about school, explaining that some ‘children are sometimes picked on, threatened, hit or beaten by other children’ and that ‘these children can be in [their] class or [they] can meet them in the school playground or on [their] way to school’. Youth were asked to report on their experience of direct and indirect victimization in the past 6 months *via* a 4-point Likert-scale (0 = never; 1 = 1-3 times; 2 = 4 or more times; 3 = at least once per week). Direct victimization was assessed *via* 5 items (hitting or beating; threatening or blackmailing; taking personal belongings; tricking in a nasty way; calling bad/nasty names), while indirect victimization was assessed *via* 4 items (telling lies or nasty things about them; spoiling games; excluding to upset them; pressuring them to do things they don’t want to). Victimization was coded present if youth reported any of the bullying behavior occurring at least four or more times in the past 6 months. Youth were also asked about peer bullying perpetration, using the same method described above. Peer bullying groups (victims, bully-victims, bullies) were constructed using a cut-off of frequent peer bullying experiences (>4 times in the last 6 months) using the algorithm as described above for sibling bullying (9).

### Outcomes

Outcome measures were collected during two focus clinic sessions. Depression and anxiety were derived *via* the Computerized Interview Schedule — Revised (CIS-R), a self-administered computerized interview which derives a diagnoses based on algorithms according to the International Classification of Diseases, 10^th^ Revision (ICD-10) criteria for depression and anxiety disorder within the time frame of the past 2 weeks. The computerized version of the CIS-R has been found to show close agreement with the interviewer administered version (36, 37). Questions on suicidal ideation and self-harm were based on items used in the Child and Adolescent Self-Harm in Europe (CASE) study (38).

#### Depression

Depression at 18 and 24 years was assessed according to the severity (frequency, duration and unpleasantness) of the core symptoms of depressive disorders (depression, depressive thoughts, fatigue, sleep and concentration problems) experienced in the last 2 weeks, using algorithms based on ICD-10 criteria, classifying individuals as presenting a mild, moderate or severe diagnosis of depression. A binary variable was constructed (0 = no depression; 1 = mild, moderate or severe depression).

#### Anxiety

Anxiety at 18 years was assessed according to presence of either one of the following anxiety disorders: generalized anxiety disorder, social phobia, specific/isolated phobia, panic disorder or agoraphobia as derived by algorithms based on ICD-10 criteria of symptoms of anxiety disorders in the last 2 weeks. Anxiety at 24 years was assessed in the same way, with the exception of agoraphobia which was excluded due to the small number of those diagnosed (<5). Positive responses were collapsed into a binary variable (0 = no anxiety; 1 = at least one anxiety disorder), one for anxiety disorder at 18 years and one for anxiety disorder at 24 years.

#### Suicidal Ideation

Suicidal ideation was assessed at 24 years only *via* 1 item asking participants whether they had ever had thoughts about killing themselves at some point during their lifetime (39). Responses were coded as a binary variable (0 = not present; 1 = present).

#### Self-Harm

Lifetime history of self-harm was assessed at 18 years and 24 years. At 18 years self-harm was assessed using a binary variable (0 = no self-harm; 1 = self-harm) coded from responses to the following question: “Have you ever hurt yourself on purpose in any way (e.g., by taking an overdose of pills, or by cutting yourself)?” (16). Self-harm at 24 years was assessed *via* 3 items. Participants were first asked whether they had ever hurt themselves on purpose in any way. Those who responded positively to that question were classified as presenting a history of self-harm. Two additional questions were then asked in order to differentiate between those who had self-harmed with suicidal intent from those who had self-harmed without suicidal intent at some point during their lifetime (39, 40). Epidemiological studies have found that there are differences in prevalence, frequency as well as contributing risk factors between self-harm that occurs with an intention to die and self-harm that occurs without an intention to die (40, 41). In order to differentiate between these two forms of self-harm we constructed two binary variables: one reflecting suicidal self-harm (0 = not present; 1 = present) and one reflecting non-suicidal self-harm (0 = not present; 1 = present).

### Potential Confounders

Precursors were included as potential confounders if they were assessed prior 8 years of age (self-reported onset of sibling bullying victimization and perpetration).

Maternal depression was assessed at 32 weeks’ gestation in pregnancy *via* the Edinburgh Postnatal Depression Scale (42). Birth order was assessed at 7 years and used as a binary variable (0 = later born; 1 = first-born). Internalizing and externalizing problems were assessed *via* the Strengths and Difficulties Questionnaire (SDQ; 43) according to maternal reports when the study child was 7 years. The emotional subscale was used in order to assess internalizing problems, whereas the conduct problems and hyperactivity scales were used in order to assess externalizing problems. Previous mental health was assessed *via* the Development and Well-being Assessment (DAWBA; 44) based on parental reports when children were 7 years old. Children were coded as presenting no DSM-IV Axis I diagnoses (N = 5,697, 90.4%) or one or more DSM-IV Axis I diagnoses of attention deficit hyperactivity disorder, conduct disorder, oppositional defiant disorder, depression, or anxiety (45). Peer bullying at 8 years was assessed *via* the Bullying and Friendship Interview Schedule (35) using the same cut-off criteria as described for peer bullying at 12 years above. Maltreatment was assessed *via* maternal reports and coded as present if mothers reported children’s experience of physical or sexual abuse, or if their child had been put into care across three timepoints (18, 30 or 42 months) (16). Domestic violence was assessed *via* maternal reports and coded as present if mothers reported any physical or emotional cruelty from their partner across four timepoints (8, 21, 33 or 47 months) (46).

### Statistical Analysis

All analysis was conducted using STATA version 14.0. χ^2^ analysis were first performed in order to examine whether there were any sex differences across sibling bullying victimization and perpetration. Further χ^2^ tests were then performed in order to test for dependency between sibling and peer bullying involvement across the different roles (**Table 3**).

A set of binary logistic regression analyses were carried out in order to assess whether sibling bullying roles (non-involved, victim, bully-victim, bully) were associated with mental health outcomes. In order to assess the crude associations between sibling bullying roles and depression, anxiety and self-harm at 18 years (late adolescence) a set of three binary logistic regression models were run (see **Table 4**). In order to assess the crude associations between sibling bullying roles and depression, anxiety, suicidal ideation, and self-harm (with and without suicidal intention) at 24 years (early adulthood) a set of five binary logistic regression models were run (see **Table 5**). Odds ratios (OR) and 95% confidence intervals (CI) are reported.

Next, we tested whether bullying victimization across multiple contexts (home and school) would result in a cumulative risk of a diagnosis of depression, anxiety disorders, suicidal ideation or self-harm in early adulthood (see **Table 5**). Sibling or peer bullying victimization was considered present if children qualified as either a victim or a bully-victim. An ordinal variable was created for sibling and peer bullying victimization (non-involved, either or both). Separate binary logistic regression models were run for each one of the outcome measures, reflecting the crude associations.

Bonferroni corrections were applied across our models in order to account for multiple testing and guard against type I error (47).

In order to account for missing data by attrition, we applied fully conditional specification equations as implemented in Multiple Imputations by Chained Equations algorithm (MICE) in STATA 14 (“mi impute” command). An averaged parameter estimate of 60 imputed datasets was used according to Rubin’s rule (48). In order to improve our model, we included sociodemographic variables as auxiliary variables, as these have been associated with missingness in ALSPAC. We then re-ran our initial binary logistic regression models using the imputed dataset, this time including all confounding variables. We further omitted participants who presented any psychiatric diagnosis (DSM-IV Axis I diagnoses according to DAWBA) prior to our measure of exposure (sibling bullying) throughout our regression models in order to additionally guard for reverse causality. Adjusted analyses are found within the same tables as the unadjusted analyses, in order to allow for direct comparisons (**Tables 5** and **6**). We were able to impute up to the same starting sample as seen in our crude logistic regression models.

## Results

### Prevalence and Characteristics of Sibling and Peer Bullying

Children reported the onset of sibling bullying victimization (*M =* 8.3, *SD =* 2.51) and perpetration (*M = *8.7, *SD =* 2.51) around 8 years of age. Females were more often victimized (55.84%), χ^2^(1) = 6.32, *p = *0.012, whereas males were more often the perpetrators of sibling bullying (51.2), χ^2^(1) = 13.31, *p* < 0.001. The most frequent types of sibling bullying behavior were either physical (hitting, kicking, pushing or shoving) or psychological (name calling or making fun of). Property-based and social forms of sibling bullying were less common (see **Table 1**).

**Table 1:** Frequencies of different types of sibling bullying victimization and perpetration behaviors.

Type of bullying^a^	Victimization N (% of total sample)	Perpetration N (% of total sample)
Hit, kicked, pushed, or shoved	1,015 (31.0)	760 (27.4)
Possessions damaged or taken	210 (6.4)	65 (2.4)
Called names	1,357 (41.3)	945 (33.9)
Made fun of	1,021 (31.3)	562 (20.5)
Ignored or left out of games or social groups	357 (11.0)	227 (8.2)
Told lies or spread rumors	270 (8.3)	54 (2.0)
Bullied in another way	126 (4.3)	42 (1.7)

a All types of sibling bullying are considered present if reported at least once a week.

Sibling bullying (31.2%) was overall reported more frequently compared to peer bullying (27.6%). Most children involved in sibling bullying were bully-victims (N = 914, 13.4%) or victims (N = 878, 12.8%), with bullies making up the smallest group (N = 342, 5%). In contrast, most children involved in peer bullying were victims (N = 1,166, 17.7%), followed by bully-victims (N = 459, 7%) and bullies (N = 192, 2.9%).

**Table 2** provides an overview of the prevalence of our mental health outcomes across our overall sample as well as specific to the sibling bullying and peer bullying groups.

**Table 2:** Prevalence of mental health outcomes at 24 years according to sibling and peer bullying roles at 12 years (in percentage).

		Depression	Anxiety	Suicidal ideation	Non-suicidal self-harm	Suicidal self-harm
**Siblings**	Non-involved	8.5	8.9	26.6	15.1	3.9
	Victim	10.0	8.7	35.4	16.2	8.3
	Bully-victim	15.1	12.5	35.7	16.1	4.9
	Bully	9.0	7.1	31.0	14.0	2.0
	Overall sample	9.6	9.3	29.0	15.3	4.5
**Peers**	Non-involved	8.5	8.0	24.7	13.8	3.7
	Victim	10.8	11.6	35.9	20.2	8.0
	Bully-victim	14.2	16.0	44.7	19.4	11.2
	Bully	16.9	10.2	44.1	25.4	8.5
	Overall sample	9.5	9.2	28.5	15.6	5.1

Non-Involved, Youth reporting no frequent* victimization or perpetration; Victims, Youth reporting frequent victimization only; Bully-Victims, Youth reporting both frequent victimization and perpetration; Bullies, Youth reporting frequent perpetration only.

*Frequent, At least once a week in the past 6 months.

### Association Between Sibling and Peer Bullying

Sibling and peer bullying were significantly associated (χ^2^(1) = 179.27, *p* < 0.001). Multinomial logistic regression analyses revealed a homotypic relationship between sibling and peer bullying. Sibling victim and bully-victim status predicted peer victim status; Sibling bully and bully-victim status was associated with peer bully status; Involvement in any kind of sibling bullying role predicted peer bully-victim status (see **Table 3**).

**Table 3:** Associations between sibling and peer bullying at 12 years.

RR (95% CI)	Sibling bullying status					
Peer bullying status	Victim	*p* Value	Bully-Victim	*p* Value	Bully	*p* Value
Victims	**1.43 (1.14–1.78)**	**0.002**	**1.85 (1.49–2.28)**	0.000	1.39 (0.99–1.95)	0.056
Bully-victim	**2.14 (1.52–3.02)**	**0.000**	**4.78 (3.62–6.32)**	0.000	**3.32 (2.16–1.63)**	**0.000**
Bully	1.47 (0.87–2.48)	0.149	**2.41 (1.54–3.79)**	0.000	**3.14 (1.77–5.58)**	**0.000**

RR, Relative risk ratios; CI, Confidence intervals.

Victims, Youth reporting victimization only; Bully-Victims, Youth reporting both victimization and perpetration; Bullies, Youth reporting perpetration only.

Bold = p < .05.

### Associations With Depression, Anxiety, Suicidal Ideation, and Self-Harm

Youth involved as sibling bully-victims in middle childhood were at increased risk for clinical depression (OR = 2.23; 95% CI, 1.54–3.22) and anxiety (OR = 1.72; 95% CI, 1.22–2.41), as well as self-harm (OR = 2.06; 95% CI, 1.7–2.89) in late adolescence at 18 years. Moreover, youth involved as sibling bully-victims in middle childhood were nearly twice the odds of being diagnosed with depression in early adulthood at 24 years (OR = 1.91; 95% CI, 1.33–2.72). Youth experiencing sibling victimization, either as a victim or a bully-victim were further at an increased risk of suicidal ideation (victims: OR = 1.52; 95% CI, 1.16–1.98; bully-victims: OR = 1.54; 95% CI, 1.19–1.99) as well as suicidal self-harm (OR = 2.20, 95% CI, 1.36–3.58) in early adulthood. Associations remained and similar in strength once all childhood confounders were included into the imputed and adjusted model (see **Tables 4** and **5**).

**Table 4:** Associations between sibling bullying status groups at 12 years and depression, anxiety and self-harm at 18 years.

	Sibling bullying status						
Outcome OR (95% CI)	Non-involved	Victim	*p* value	Bully-victim	*p* value	Bully	*p* value
N = 2,802							
**Depression**							
Unadjusted	Reference	1.62 (1.07–2.45)	0.022	**2.23 (1.54–3.22)**	**0.000**	0.77 (0.33–1.78)	0.539
Imputed adjusted	Reference	1.56 (1.03–2.37)	0.038	**2.06 (1.41–3.01)**	**0.000**	0.92 (0.39–2.16)	0.854
**Anxiety**							
Unadjusted	Reference	1.04 (0.69–1.56)	0.853	**1.72 (1.22–2.41)**	**0.002**	0.60 (0.28–1.31)	0.198
Imputed adjusted	Reference	0.99 (0.66–1.49)	0.959	**1.57 (1.11–2.23)**	**0.011**	0.71 (0.32–1.56)	0.389
**Self-Harm**							
Unadjusted	Reference	1.38 (0.94–2.03)	0.103	**2.06 (1.47–2.89)**	**0.000**	0.88 (0.44–1.76)	0.713
Imputed adjusted	Reference	1.29 (0. 87–1.91)	0.201	**1.85 (1.31–2.61)**	**0.001**	1.08 (0.53–2.20)	0.834

OR, Odds ratio; CI, Confidence intervals.

Bold = p < 0.017 (Bonferroni correction).

Confounders included in imputed adjusted analysis: sex, maternal depression, internalizing and externalizing problems, peer bullying victimization and perpetration, maltreatment, domestic violence.

Children presenting any psychiatric diagnosis at 7 years were excluded.

Non-Involved, Youth reporting no frequent* victimization or perpetration; Victims, Youth reporting frequent victimization only; Bully-Victims, Youth reporting both frequent victimization and perpetration. Bullies, Youth reporting frequent perpetration only.

*Frequent, At least once a week in the past 6 months.

**Table 5:** Associations between sibling bullying status groups at 12 years and depression, anxiety suicidal ideation and self-harm at 24 years.

	Sibling bullying status						
Outcome OR (95% CI)	Uninvolved	Victim	*p* value	Bully-victim	*p* value	Bully	*p* value
**Depression**							
N = 2,373							
Unadjusted	Reference	1.19 (0.78–1.81)	0.423	**1.91 (1.33–2.72)**	**0.000**	1.06 (0.52–2.15)	0.870
Imputed adjusted	Reference	1.19 (0.78–1.83)	0.421	**1.78 (1.23–2.58)**	**0.002**	1.14 (.55–2.36	0.719
**Anxiety**							
N = 2,359							
Unadjusted	Reference	0.97 (0.62–1.50)	0.874	1.46 (1.00–2.13)	0.052	0.78 (0.35–1.50)	0.526
Imputed adjusted	Reference	0.92 (0.59–1.45)	0.732	1.33 (.90–1.96)	0.152	0.86 (9.38–1.92)	0.712
**Suicidal ideation**							
N = 2, 372							
Unadjusted	Reference	**1.52 (1.16–1.98)**	**0.002**	**1.54 (1.19–1.99)**	**0.001**	1.24 (0.80–1.92)	0.331
Imputed adjusted	Reference	**1.47 (1.12–1.92)**	**0.005**	1.40 (1.07–1.82)	0.013	1.20 (0.76–1.88)	0.432
**Non-suicidal self-harm**							
N = 2,372							
Unadjusted	Reference	1.09 (0.78–1.53)	0.621	1.08 (0.78–1.51)	0.642	0.92 (0.51–1.64)	0.770
Imputed adjusted	Reference	1.05 (0.74–1.59)	0.774	0.99 (0.70–1.40)	0.960	1.13 (0.62–2.06)	0.690
**Suicidal self-harm**							
N = 2, 372							
Unadjusted	Reference	**2.20 (1.36–3.58)**	**0.001**	1.27 (0.71–2.25)	0.418	0.50 (0.12–2.07)	0.337
Imputed adjusted	Reference	**2.19 (1.34–3.59)**	**0.002**	1.22 (0.68–2.19)	0.498	0.57 (0.14–2.39)	0.441

OR, Odds ratio; CI, Confidence intervals.

Bold = p < 0.010 (Bonferroni correction).

Confounders included in imputed adjusted analysis: sex, birth order, maternal depression, internalizing and externalizing problems, peer bullying victimization and perpetration, maltreatment, domestic violence.

Children presenting any psychiatric diagnosis at 7 years were excluded.

Non-Involved, Youth reporting no frequent* victimization or perpetration; Victims, Youth reporting frequent victimization only; Bully-Victims, Youth reporting both frequent victimization and perpetration; Bullies, Youth reporting frequent perpetration only.

*Frequent, At least once a week in the past 6 months.

Youth who were victimized by their siblings as well as their peers were at a two-fold increased risk of depression (OR = 1.97; 95% CI, 1.21-3.21) and suicidal ideation (OR = 2.37, 95% CI, 1.69-3.33) as well suicidal self-harm (OR = 3.46, 95% CI, 1.92-6.25). The odds of depression, suicidal ideation, and self-harm behavior were only slightly attenuated once all confounders were included in the imputed and adjusted model (see **Table 6**). A linear trend was observed for depression, suicidal ideation, and suicidal self-harm, suggestive of a cumulative relationship between multiple victimization (home and school environment) and increased likelihood of clinical depression or engagement in suicidal ideations self-harm.

**Table 6:** Individual and cumulative effects of sibling and peer bullying at 12 years and depression, anxiety suicidal ideation and self-harm at 24 years.

	Sibling and peer bullying status						
Outcome OR (95% CI)	Uninvolved	Either	*p* value	Both	*p* value	Linear trend	*p* value
**Depression**							
N = 2,117							
Unadjusted	Reference	**1.50 (1.09–2.05)**	**0.012**	**1.97 (1.21–3.21)**	**0.006**	**1.43 (1.16–1.78)**	**0.001**
Imputed adjusted	Reference	**1.45 (1.05–1.99)**	0.024	1.90 (1.15–3.13)	0.012	**1.40 (1.12–1.75)**	**0.003**
**Anxiety**							
N = 2,105							
Unadjusted	Reference	**1.85 (1.35–2.53)**	**0.000**	1.78 (1.05–3.01)	0.032	**1.49 (1.20–1.85)**	**0.000**
Imputed adjusted	Reference	**1.73 (1.26–2.39)**	**0.001**	1.60 (0.94–2.75)	0.085	**1.41 (1.13–1.76)**	**0.003**
**Suicidal ideation**							
N = 2,118							
Unadjusted	Reference	**1.61 (1.31–1.97)**	**0.000**	**2.37 (1.69–3.33)**	**0.000**	**1.57 (1.36–1.81)**	**0.000**
Imputed adjusted	Reference	**1.54 (1.25–1.90)**	**0.000**	**2.18 (1.54–3.07)**	**0.000**	**1.50 (1.30–1.74)**	**0.000**
**Non-suicidal self-harm**							
N = 2,117							
Unadjusted	Reference	1.36 (1.06–1.75)	0.016	1.32 (0.85–2.05)	0.211	1.23 (1.03–1.47)	0.024
Imputed adjusted	Reference	1.31 (1.01–1.69)	0.041	1.27 (0.81–1.98)	0.303	1.19 (0.99–1.44)	0.060
**Suicidal self-harm**							
N = 2,117							
Unadjusted	Reference	**1.79 (1.15–2.79)**	**0.010**	3.46 (1.92–6.25)	**0.000**	**1.85 (1.39–2.45)**	**0.000**
Imputed adjusted	Reference	1.77 (1.13–2.78)	0.012	3.47 (1.90–6.34)	**0.000**	**1.84 (1.38–2.46)**	**0.000**

OR, Odds ratio; CI, Confidence intervals.

Bold = p < 0.010 (Bonferroni correction).

Confounders included in imputed adjusted analysis: sex, birth order, maternal depression, internalizing and externalizing problems, psychiatric diagnosis, peer bullying victimization and perpetration, maltreatment, domestic violence.

Children presenting any psychiatric diagnosis at 7 years were excluded.

Non-Involved, Youth reporting no frequent* victimization or perpetration; Victims, Youth reporting frequent victimization only; Bully-Victims, Youth reporting both frequent victimization and perpetration; Bullies, Youth reporting frequent perpetration only.

*Frequent, At least once a week in the past 6 months.

## Discussion

This study finds that youth involved in sibling victimization were associated with an increased risk of clinical depression, anxiety and self-harm behavior in late adolescence, as well as clinical depression, suicidal ideation, and suicidal self-harm in early adulthood, even after accounting for a range of potential confounders. Moreover, concurrent sibling and peer bullying are found to overlap in a homotypic role specific manner. The effects of sibling and peer bullying were found to be cumulative for depression, suicidal ideation, and suicidal self-harm. Those bullied at home and by peers had double the odds of developing clinical depression and consider suicide and triple the odds to have self-harmed with a suicidal intention by early adulthood. In contrast, anxiety disorder appeared to be particularly associated with peer rather than sibling bullying.

Previous studies investigating the relationship between sibling bullying and depression, anxiety, suicidal ideation, or self-harm have been limited to exploring just victimization or looked at involvement in sibling bullying in any role (6, 15, 16). This study extends prior work and adds to the literature by presenting for the first time evidence for a role specific relationship between sibling bullying involvement and depression, anxiety and self-harm related behavior. Our findings show that the strength of association is stronger for some roles than others, suggesting role specific effects for sibling victims and bully-victims in relation to depression, anxiety, suicidal ideation, and self-harm. It was found that youth involved as sibling bully-victims are associated with an increased risk of clinical anxiety and self-harm behavior in late adolescence and clinical depression in both late adolescence as well as early adulthood. On the other hand, youth involved as sibling victims only were associated with an increased risk of suicidal ideation and suicidal self-harm in early adulthood. These findings are partially in line with work by Bar-Zomer and Klomek (15) who reported involvement in any sibling bullying as a correlate of both clinical depression and suicidal ideation. It is however not possible to parcel out whether our results match those of Bar-Zomer and Klomek (15) in a role specific manner, as they did not tease out sibling victimization and perpetration from one another. Similarly, Coyle et al. (6) report a concurrent relationship between sibling victimization with depression and anxiety, while Bowes et al. (16) identified a prospective link between frequent sibling victimization with clinical depression and as self-harm.

Our results suggest that the link between sibling bullying in middle childhood with depression and self-harm related behavior may persist into early adulthood. Contrary to this, the association between sibling bullying and anxiety appears to be limited to late adolescence. In our adjusted models we found that pre-existing internalizing problems explained some of the observed variance, suggesting that the association between bullying and anxiety disorders may rather be understood as a function of pre-existing internalizing problems as opposed to a causal effect of sibling victimization per se. Anxiety disorders are furthermore reported as an early onset disorder (48), hence it may be that the exclusion of children with a psychiatric diagnosis in early childhood resulted in the desisting association between sibling bullying and clinical anxiety in early adulthood.

Nevertheless, our overall findings in relation to differential sibling bullying group outcomes resonate well within the peer literature. Peer victimization has been proposed as a robust contributing factor towards the development of internalizing problems (12), with those children falling into the bully-victim group at the greatest risk for poor mental health outcomes (7, 9,, 25), as mirrored by our results.

In contrast, prospective studies have previously reported for peer bullying (7, 49) that those who are bullies were at no increased risk for emotional disorders, self-harm or suicidal ideation. This is consistent with an evolutionary model of bullying that suggests bullying perpetration as an evolutionarily adaptive behavior (50). Recent evidence from ALSPAC has shown that sibling bullying perpetration was best predicted by structural family characteristics (51) including larger households with more children, being older and male, all of which are factors contributing towards a heightened competition of resource availability within the family system. These findings underline that aggression or fighting may be utilized as a mechanism for children to secure resources and restore social dominance (52) within their social group (family or peer group), thereby gaining desired outcomes including affection, attention or material goods within the family system or social status and mating opportunities within the peer context (53, 54). Along these lines, bullying perpetration may even act protective against adverse health outcomes, as mirrored by our results in which youth who acted as bullies were no more likely than non-involved youth to develop depression, anxiety, suicidal ideations, or self-harm behavior.

Findings from this study further demonstrate that sibling bullying victimization in middle childhood is an independent risk factor towards the development of clinical depression, suicidal ideation, and suicidal self-harm in early adulthood above and beyond the influence of peer bullying as well as other early childhood predictors of poor mental health, parallel to previous work on the link between sibling victimization and internalizing problems (6, 16). This evidence strongly suggests that sibling bullying should not be normalized as a harmless rite of passage. It further stresses that sibling bullying should be considered as a unique contributing factor towards adverse mental health and wellbeing, beyond peer bullying and must therefore be appropriately addressed by families and practitioners. These unique effects were found despite a significant association between sibling and peer bullying. The cross-over between sibling and peer bullying was found to be homotypic, i.e. role specific consistent with previous reports (21, 26, 27, 29). In other words, children who were sibling victims or bully-victims at home were more likely peer victims in school, while sibling bullies and bully-victims at home were more often peer bullies at school.

Finally, a dose–response effect relationship of exposure to victimization across multiple contexts and mental health outcomes was found. Youth who were victimized by their siblings and their peers displayed higher odds of adult mental health problems across the domains of clinical depression, suicidal ideation, and suicidal self-harm, as opposed to youth involved in either sibling or peer victimization alone. Unlike previous work from the peer literature, suggesting bullying victimization as a common risk factor of both suicidal and non-suicidal self-harm (40), our findings suggest sibling bullying as well as poly-victimization across the sibling and peer context as specific risk factors more strongly associated with suicidal self-harm. These findings extend findings from cross-sectional studies of sibling and peer bullying and emotional problems (19, 20, 29) that this cumulative effect is confirmed using clinical diagnosis and longitudinal data, affecting depression suicidal ideation, and self-harm up to 12 years later. These findings further indicate that bullying as a trauma is most harmful when youths experience this at home as well as at school. For the affected youth it means that they have no safe place to escape to and this increases the risk of serious mental health problems such as suicidal ideation, self-harming, and depression. Peer and sibling bullying are traumas that should be considered at par with traumas such as physical or sexual abuse (14). However, as both peer and sibling bullying are more frequent than abuse and maltreatment by adults (2, 3, 14), their impact on population health may exceed those of adult maltreatment (9).

While we did find some evidence of a linear trend between poly-victimization and clinical anxiety in early adulthood, we did not find a cumulative effect of sibling and peer victimization when explored as an ordinal term. One reason for this may be that child individual differences may have accounted for a large proportion of the observed variance, as illustrated for child internalizing problems in our adjusted imputed model. In the peer literature, there is indeed evidence suggesting that children who suffer from internalizing disorders are more likely to become victimized (8). Alternatively, it may also be that peer bullying plays a more substantial role in the development of anxiety disorders (9, 55) compared to sibling bullying, as reflected in our findings. Lastly, it is possible that anxiety may not persist beyond late adolescence.

### Strengths and Weaknesses

This study has a number of strengths. First, the longitudinal nature of our study design allowed us to prospectively assess a large number of potential confounding variables from pregnancy until childhood, thereby decreasing measurement error and bias and increasing the confidence in a causal relationship between our exposure and outcome measures. Additionally, excluding children who were classified as presenting a psychiatric disorder in early childhood, prior to our measure of exposure (sibling bullying), minimized reverse causation and thereby increases confidence in our findings. Moreover, we were able to prospectively explore mental health outcomes up to 12 years after the assessment of sibling bullying, allowing us to test whether the experience of sibling bullying could predict depression, anxiety and self-harm related thoughts and behaviors into early adulthood. This study also utilized Bonferroni correction across our regression models in order to guard against type I error, thereby making our analysis more conservative and in turn increasing the confidence in our findings.

There are also limitations to this study. Longitudinal data like ALSPAC is naturally prone to missing data over a 24 year study period, allowing for the possibility of attrition bias. However, there has been evidence demonstrating that accurate predictions are not compromised even in the face of selective dropout (56). Nonetheless, we additionally addressed the possibility of attrition bias by performing multiple imputations, thereby accounting for missing data and allowing us to impute up to our initial sample size. Another weakness of our study is that sibling bullying was measured at a single time point. Future work should strive to include multiple measures of sibling bullying in order to allow for the exploration of dose–response effect of chronicity, as it is often done within the peer literature (57). Nevertheless, our study shows that even a single measure of sibling bullying was sufficient to predict clinical depression and suicidal ideation, stressing the importance of considering sibling bullying as an additional specific risk factor towards the development of mental health problems. Finally, it should be noted that our exposure and outcome measures were assessed* via *self-report only, which may have biased our results. In the sibling literature, sibling bullying has been found to occur behind closed doors with parents often unaware of this behavior (1). Thus, self-reported sibling bullying may provide more accurate measures as opposed to parental reports. The use of the self-administered computerized CIS-R has further been shown to be a valid and unbiased measure of psychiatric disorder when compared to assessments administered through a human interviewer (58). Nonetheless, future studies should aim to include multi-rater reports of bullying and mental health outcomes in order to test whether associations will persist in a similar strength and to further reduce any bias that may result from youth’s perception of bullying on mental health outcomes.

### Conclusion

Our results have important practical and clinical implications. Firstly, it is essential for parents and health care professionals to be made aware that sibling victimization in childhood may result in lasting mental health consequences. Secondly, the effects of sibling bullying are at par with those of peer bullying where there is now convincing evidence for the detrimental effect on mental health (1, 10). Thirdly, those bullied at home by siblings are more likely to be involved in bullying at school. For the victims this means that they have no safe place to escape bullying and torment. Parents in particular may benefit from psychoeducational programs that help them recognize early warning signs of sibling bullying and support them towards intervening effectively in order to improve and foster long-lasting positive sibling relationships (59). Health professionals working with children and families on the other hand, should be encouraged to regularly enquire about sibling and peer bullying experiences, as these may be early warning signs of poor mental health and wellbeing (60). Finally, there is a need for the development, implementation and assessment of intervention studies that are specifically tailored towards reducing sibling bullying, as there are currently no well tested programs available (61, 62). Such interventions hold promise for alleviating a range of consequent negative outcomes, including the prevention of peer bullying, which appears to be contingent on early bullying experiences in the home environment.

## Data Availability

The datasets analyzed for this study can be requested from ALSPAC. Please note that the study website contains details of all the data; this is available through a fully searchable data dictionary and variable search tool (http://www.bristol.ac.uk/alspac/researchers/our-data/).

## Ethics Statement

The studies involving human participants were reviewed and approved by ALSPAC Ethics and Law Committee (IRB No. 00003312) and the local research ethics committees (Bristol and Weston Health Authority, Southmead Health Authority, and Frenchay Health Authority). Written informed consent to participate in this study was provided by the participants’ legal guardian/next of kin.

## Author Contributions

SD and DW contributed towards the design and interpretation of the data. SD was responsible for the analysis of the data and drafting of the work. DW provided a critical review and revisions of the work. SZ, MH, and JH contributed toward the acquisition of the data and contributed toward the final revisions.

## Funding

The UK Medical Research Council and Wellcome (Grant ref: 102215/2/13/2) and the University of Bristol provide core support for ALSPAC. This publication is the work of the authors and SD and DW will serve as guarantors for the contents of this paper.

SZ and MH are supported by the NIHR Biomedical Research Centre at University Hospitals Bristol NHS Foundation Trust and the University of Bristol. The views expressed in this publication are those of the author(s) and not necessarily those of the NHS, the National Institute for Health Research or the Department of Health and Social Care.

A comprehensive list of grants funding is available on the ALSPAC website (http://www.bristol.ac.uk/alspac/external/documents/grant-acknowledgements.pdf); This research was specifically funded by the Wellcome Trust and MRC (Grant ref. 076467/Z/05/Z) in relation to all of the depression, anxiety and self-harm measures collected at the Focus@24 clinic data.

## Conflict of Interest Statement

The authors declare that the research was conducted in the absence of any commercial or financial relationships that could be construed as a potential conflict of interest.
